# 重建参数对CT肺结节容积测量的影响

**DOI:** 10.3779/j.issn.1009-3419.2012.02.02

**Published:** 2012-02-20

**Authors:** 荣荣 杨, 铁链 于, 颖 王, 卿 王

**Affiliations:** 300052 天津，天津医科大学总医院放射科 Department of Radiology, General Hospital of Tianjin Medical University, Tianjin 300052, China

**Keywords:** 计算机体层摄影, 肺结节, 容积测量, 算法, 层厚, Computed tomography, Lung nodule, Volumetry, Algorithms, Slice thickness

## Abstract

**背景与目的:**

已有的研究证明：CT肺结节容积测量能够敏感反映结节容积的微小变化，在未定性肺结节的随访观察及良恶性鉴别方面具有重要应用前景。本研究旨在评估不同重建算法和层厚对CT肺结节容积测量的影响。

**方法:**

对2009年12月-2011年8月在天津医科大学总医院行未定性肺结节CT随访研究的30例患者的CT图像进行后处理分析。共获取52个肺结节，应用3种不同的算法（骨密度算法、标准算法及肺算法）及层厚（0.625 mm、1.25 mm、2.5 mm）进行9次重建。由一位放射科医师应用肺结节容积分析软件进行容积测量。应用重复测量多元方差分析、相关分析及*Bland*-*Altman*法评价结节直径、算法、层厚对容积测量的影响。

**结果:**

不同重建算法（*F*=13.6, *P*＜0.001）、层厚（*F*=4.4, *P*=0.02）条件下测量所得的结节容积之间具有统计学差异。肺结节9次测量所得容积的变异系数和结节直径之间呈负相关（*r*=-0.814, *P*＜0.001）。应用2.5 mm层厚时，结节容积比1.25 mm及0.625 mm层厚的一致性差，1.25 mm和0.625 mm层厚在采用骨算法时一致性最好。

**结论:**

不同重建算法及层厚对肺结节容积测量有影响，尤其是直径较小的结节。在未定性肺结节尤其是肺小结节的随访过程中建议应用相同的重建参数。

## Abstract (en)

**Background and objective:**

It has been proven that volumetric measurements could detect subtle changes in small pulmonary nodules in serial CT scans, and thus may play an important role in the follow-up of indeterminate pulmonary nodules and in differentiating malignant nodules from benign nodules. The current study aims to evaluate the effects of different reconstruction parameters on the volumetric measurements of pulmonary nodules in chest CT scans.

**Methods:**

Thirty subjects who underwent chest CT scan because of indeterminate pulmonary nodules in General Hospital of Tianjin Medical University from December 2009 to August 2011 were retrospectively analyzed. A total of 52 pulmonary nodules were included, and all CT data were reconstructed using three reconstruction algorithms and three slice thicknesses. The volumetric measurements of the nodules were performed using the advanced lung analysis (ALA) software. The effects of the reconstruction algorithms, slice thicknesses, and nodule diameters on the volumetric measurements were assessed using the multivariate analysis of variance for repeated measures, the correlation analysis, and the *Bland*-*Altman* method.

**Results:**

The reconstruction algorithms (*F*=13.6, *P* < 0.001) and slice thicknesses (*F*=4.4, *P*=0.02) had significant effects on the measured volume of pulmonary nodules. In addition, the coefficients of variation of nine measurements were inversely related with nodule diameter (*r*=-0.814, *P* < 0.001). The volume measured at the 2.5 mm slice thickness had poor agreement with the volumes measured at 1.25 mm and 0.625 mm, respectively. Moreover, the best agreement was achieved between the slice thicknesses of 1.25 mm and 0.625 mm using the bone algorithm.

**Conclusion:**

Reconstruction algorithms and slice thicknesses have significant impacts on the volumetric measurements of lung nodules, especially for the small nodules. Therefore, the reconstruction setting in serial CT scans should be consistent in the follow-up of indeterminate pulmonary nodules, more importantly for the small nodules.

随着多排螺旋CT的广泛应用，肺结节尤其是肺小结节（直径＜1 cm的结节）被越来越多地检测出来^[1]^。现有的影像检查方法对肺小结节的定性困难，PET-CT及穿刺活检也常难达到满意效果。肺结节的生长特性成为目前临床判断其良恶性的重要指标之一，恶性结节常表现出持续快速增长的特征^[2]^。因此，随访监测结节变化，对容积增大的结节进行处理，实现早诊早治已经成为临床常用的策略。研究^[3, 4]^发现，基于软件的肺结节容积测量较传统人工二维测量能更敏感地检测到结节的大小变化，在肺结节随诊中具有优势并已经逐渐应用于临床实践中。然而，肺结节容积分析软件并非完美，其所测结节容积受多种因素影响，如应用的软件、不同的重建算法、层厚、结节的位置、扫描时患者的呼吸状态、操作者及对比剂的应用等。明确这些因素的影响，对于判断结节容积是否真正增长和确定容积增加的阈值具有重要意义。本研究的目的是应用肺结节容积分析软件对肺结节进行容积测量，评价不同重建算法和层厚对肺结节容积测量的影响。

## 1 资料与方法

### 1.1 临床资料

收集2009年12月-2011年8月在天津医科大学总医院行64排螺旋CT检查发现肺结节并在我院进行随访的患者。以直径＜15 mm、不与胸膜及血管相连、无钙化及空洞的实性结节作为研究对象。共有30例符合条件的患者入组，其中男性14例，女性16例，年龄范围35岁-78岁，平均年龄（58.6±8.6）岁，符合条件的结节共52个，结节平均直径（6.59±2.67）mm，直径范围3 mm-14 mm，其中直径＜5 mm的结节19个，占36.5%。

### 1.2 CT检查方法

所有扫描均在同一台螺旋CT机（GE 64排Light Speed）上进行，一次吸气后屏气完成全肺扫描，扫描参数为120 kV、300 mA、5 mm层厚，FOV 360 mm，矩阵512×512。扫描完成后，将包含结节的局部容积扫描数据用2.5 mm、1.25 mm及0.625 mm 3种层厚进行重建，每种层厚重建时分别采用3种算法：标准算法、肺算法和骨算法，共获得9组数据。

### 1.3 结节分析

所有图像分析均由一位放射科医师在GE ADW 4.2工作站上应用ALA（advanced lung analysis）软件进行。首先，在1.25 mm层厚标准算法的横断面图像上选取结节最大层面，应用电子卡尺测量并记录结节直径（最大径与其垂直短径的平均值）。然后，应用ALA软件对所获得的9组扫描数据进行结节自动容积测量，具体步骤：进入容积分析界面后，在打开的横断面图像上由观察者用鼠标单击要分析的结节，软件自动实现对结节的分割并显示出结节的容积及其在三维方向上的最大径线（图 1）。

**1:**
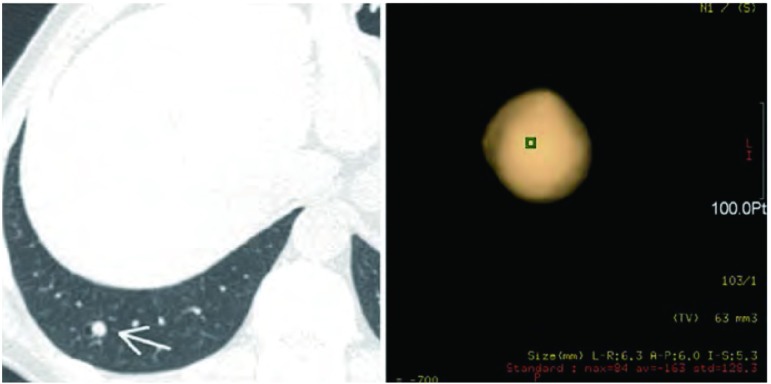
女性，56岁，查体发现右肺结节（箭），容积测量结果显示结节容积为63 mm^3^，三维方向最大径线分别为6.3 mm、6.0 mm和5.3 mm。 Female, 56 years old, a nodule (white arrow) was incidentally detected in right lower lobe during a routine physical examination. Software-generated volume of the nodule was 63 mm^3^, and the largest diameter in x, y and z is 6.3 mm, 6.0 mm and 5.3 mm respectively.

### 1.4 统计分析

应用SPSS 10.0软件进行统计分析。偏态分布的容积数据行对数转换使之符合正态分布。应用多元重复测量方差分析确定算法及层厚对结节容积测量结果的影响，在具有统计学差异基础上应用*Bonferroni*检验分别进行3种层厚及3种算法测量结果两两比较。应用*Spearman*相关分析评价容积测量总变异度和结节直径之间的关系，容积测量的总变异度用同一结节9次测量值的变异系数（coefficient of varation, CV）表示，并定义为标准差和均值的比值。应用*Bland*-*Altman*方法对＜5 mm及≥5 mm结节进行同一算法下不同层厚时容积测量结果的一致性分析，计算其相对容积差值（relative volume difference, RVD）的95%一致性区间，RVD定义为两次测量的差值与均值的比值^[5]^。*P*＜0.05为有统计学差异。

## 2 结果

### 2.1 重建参数对结节容积的影响

多元重复测量方差分析的结果显示不同重建算法（*F*=13.6, *P*＜0.001）、层厚（*F*=4.4, *P*=0.02）条件下测定的结节容积之间具有统计学差异。两两比较的结果显示标准算法与其它两种算法、2.5 mm层厚与其它两种层厚测量结果之间有统计学差异，而骨算法与肺算法、1.25 mm与0.625 mm层厚测量结果之间无统计学差异（表 1）。

**1:** 52个肺结节在不同重建算法及层厚条件下容积差异两两比较结果 Pairwise comparison of volume of 52 pulmonary nodules measured with different reconstruction algorithms and section thickness

Reconstruction paremeters		Mean volume difference^*^ (95%CI)	*P*
Algorithms	Standard *vs* lung	-7.8% (-11.9%–-3.5%)	＜0.001
	Standard *vs* bone	-6.0% (-8.8%–-3.2%)	＜0.001
	Lung *vs* bone	1.9% (-0.6%–4.5%)	0.192
Section thickness	2.5 mm *vs* 1.25 mm	4.6% (0.5%–8.8%)	＜0.001
	2.5 mm *vs* 0.625 mm	4.9% (1.6%–9.2%)	＜0.001
	1.25 mm *vs* 0.625 mm	0.3% (-1.2%–1.8%)	＜0.999
^*^These data were derived from the anti-log transformation of the log scale differences minus 100%.			

### 2.2 结节容积测量变异度和结节直径的相关分析

52个肺结节9次测量的变异系数的均值为8.8%±7.0%，相关分析的结果显示其和结节直径之间呈负相关（*r*=-0.814, *P*＜0.001）（图 2）。

**2:**
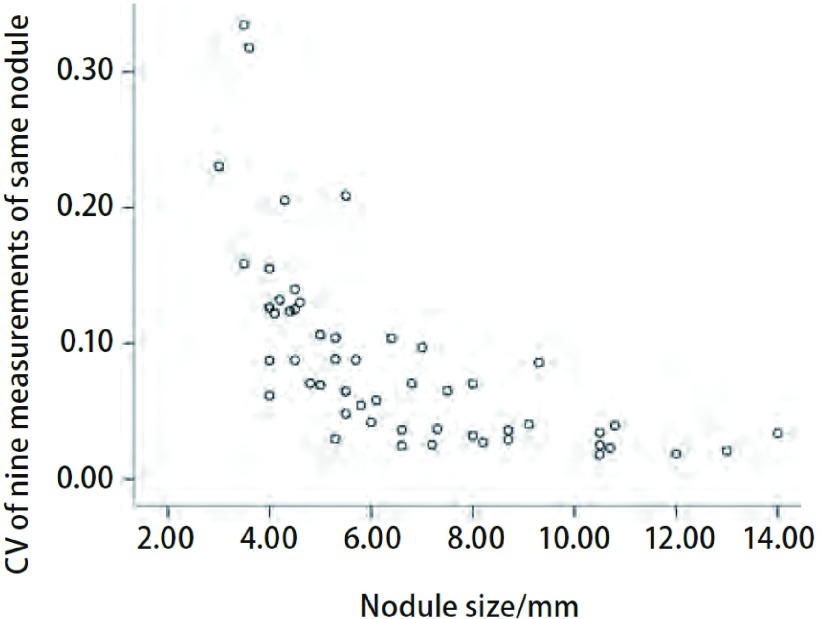
52个肺结节容积测量变异系数散点图 Scatter plot of coefficient of variation of volumetric measurement in 52 pulmonary nodules

### 2.3 容积测量的一致性分析

在3种重建算法中，除肺算法对≥5 mm结节外，2.5 mm *vs* 1.25 mm及2.5 mm *vs* 0.625 mm容积测量的95%一致性区间的范围均较1.25 mm *vs* 0.625 mm大，提示其一致性差。在不同重建条件下，≥5 mm结节的一致性均优于＜5 mm结节。采用骨算法时，1.25 mm *vs* 0.625 mm的一致性无论是对于＜5 mm还是≥5 mm结节都是最好，分别为-11.7%至13.5%及-4.7%至6.5%（表 2，图 3）。

**2:** 应用同一算法，不同层厚的52例肺结节容积测量变异度 The volumetric measurement variability of 52 pulmonary nodules with different section thickness and same reconstruction algorithm

Reconstruction algorithm	Section thickness	95% limits of agreement (RVD, %)	
		＜5 mm (*n*=19)	≥5 mm (*n*=33)
Standard	2.5 mm *vs* 1.25 mm	-39.5–43.3	-20.0–17.5
	2.5 mm *vs* 0.625 mm	-32.1–40.7	-19.1–16.3
	1.25 mm *vs* 0.625 mm	-16.0–20.7	-6.0–5.8
Lung	2.5 mm *vs* 1.25 mm	-12.2–42.8	-7.5–22.8
	2.5 mm *vs* 0.625 mm	-15.1–48.4	-9.8–21.1
	1.25 mm *vs* 0.625 mm	-22.2–24.8	-20.1–16.1
Bone	2.5 mm *vs* 1.25 mm	-38.8–52.3	-17.5–19.1
	2.5 mm *vs* 0.625 mm	-36.7–52.0	-17.3–20. 7
	1.25 mm *vs* 0.625 mm	-11.7–13.5	-4.7–6.5

**3:**
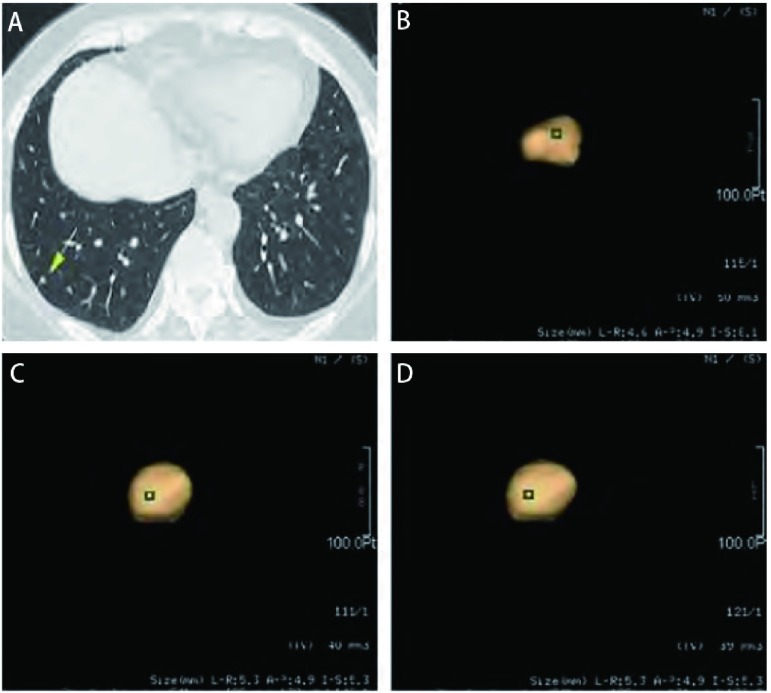
女，57岁。A：CT横断面显示右肺下叶小结节（箭），直径约4 mm；B-D：应用骨算法，不同层厚时所得结节容积；B：2.5 mm层厚，结节容积50 mm^3^；C：1.25 mm层厚，结节容积40 mm^3^；D：0.625 mm层厚，结节容积39 mm^3^。 Female, 57 years old. A: CT showed a 4 mm nodule in right lower lobe; B-D: The measured volumes using bone algorithm and different slice thickness; B: the volume is 50 mm^3^ when slice thickness is 2.5 mm; C: the volume is 40 mm^3^ when slice thickness is 1.25 mm; D: the volume is 39 mm^3^ when slice thickness is 0.625 mm.

## 3 讨论

随着多排螺旋CT的广泛使用，越来越多的肺结节，尤其是肺小结节被检测出来^[1]^。国外一项为期5年的大样本（1, 519例）肺癌筛查研究^[6]^发现：对于50岁以上的吸烟者，74%的人有至少一个肺结节，其中92%的结节为＜7 mm的小结节。如何对这些结节进行处理是目前临床所面临的一个两难的问题：一方面要早期检出恶性结节；另一方面又要避免对良性结节进行不必要的医疗处置。现有的诊断方法对于肺小结节的鉴别诊断作用有限。判断良性结节的唯一确定标准是看结节是否具有良性钙化（中心钙化、层状钙化、爆米花样钙化及弥漫多发钙化）^[6, 7]^。对于绝大多数非钙化结节良恶性的判断，CT和MR检查在目前现有技术条件下效果不够理想，PET-CT对于直径＜1 cm的结节评估良恶性的准确性也较低^[8]^。由于肺内小结节病灶较小，受呼吸运动影响大，穿刺活检难度较高，成功率较低。目前临床上最常采用的策略是随访观察，以期及时发现结节的容积变化，做出进一步处理^[9]^。因此，精确确定结节容积变化对临床肺结节处理具有重要意义。

近年来新开发的肺结节容积分析软件较传统直径测量方法能更敏感、更准确地检测到结节容积的微小变化，是一种理想的随诊方法^[3, 4]^。从理论上讲，当结节的容积增加一倍时，结节的直径才增加了26%，所以容积测量更容易及时发现结节尤其是小结节容积的变化。国外一些学者的体模研究^[10, 11]^证实应用计算机软件自动测量肺结节容积的准确性和可重复性高，是一种理想的测量方法。但是软件容积测量也有局限性，多种因素可引起测量误差，如不同的重建算法及层厚、结节的位置（与胸膜相连、与血管相连或与胸膜及血管均不相连）、患者吸气程度、不同的检查设备和分析软件、对比剂的应用与否等，均可能影响结节容积测量的准确性和可重复性。

本研究结果显示重建算法及层厚对结节的容积测量具有显著性影响；结节容积测量的变异系数与结节直径呈负相关，即随着结节直径的减小，由于层厚及重建算法不同所致的容积测量差异越大。本研究结果与国内外学者的体模及人体肺结节研究结果相似。Ravenel^[12]^利用肺结节模型研究不同大小肺结节容积测量的准确性，结果发现随着结节直径的减小，容积测量的准确性降低；层厚对于肺结节尤其是肺小结节容积测量的准确性有显著性影响。Goo等^[13]^通过模型对肺结节容积测量的研究发现：不同的重建层厚对于模型肺结节的容积测量有明显影响，结节越小容积测量变异性越大。孙海宁等^[14]^利用肺结节模型研究8种重建算法、5种重建层厚对模型肺结节容积测量准确性和可重复性测量的影响，发现肺算法误差最大，且肺算法与其他算法容积测量结果间差异有统计学意义。Wang等^[15]^应用了2种算法（软组织算法和高分辨算法）及2种层厚（1 mm、2 mm）的3种组合，研究不同重建设置对低剂量CT肺结节容积测量的影响，结果显示不同的重建层厚和算法对结节的容积测量有显著影响。Kostis等^[16]^研究结果显示随着结节直径的增加，容积测量变异度减小（2 mm-5 mm、5 mm-8 mm、8 mm-10 mm结节变异度分别是18.5%、10.6%、7.47%）。

本研究中进一步研究了在采用3种不同重建算法、3种不同重建层厚时结节容积测量结果间的一致性。我们发现当采用标准算法和骨算法时，2.5 mm与1.25 mm、0.625 mm层厚间容积测量的一致性差，1.25 mm与0.625 mm层厚间一致性相对较好；当采用骨算法时，1.25 mm与0.625 mm层厚间容积测量一致性最好。本研究结果与Petrou等^[2]^的实验结果相近，Petrou等应用一种算法（标准算法）3种层厚（5 mm、2.5 mm及1.25 mm），研究不同重建层厚对结节容积测量的影响，发现重建层厚不同对肺微小结节（3 mm-5 mm）容积测量的变异性有明显影响，结节越小容积总变异就越大。

依据上述结果，我们建议在临床随访研究中应尽可能保持重建算法和重建层厚的一致。如果遇到两次测量的重建算法或重建层厚不一致时，对于结节的容积变化要慎重分析。依据指数生长模型，肺内小结节容积倍增时间是由容积变化率及随访时间确定的。容积倍增400 d常作为恶性结节的诊断标准^[17]^，其在3个月内的容积变化率为18.6%，和层厚不同所致的容积变化一致性区间重叠。假设对一个直径＜5 mm的肺结节行3个月随访，第一次重建层厚为2.5 mm，第二次重建层厚为1.25 mm，软件容积测量结果提示结节容积在3个月内增长了18.6%，倍增时间为400 d，符合恶性结节的诊断标准，但此时的容积变化完全可能是由层厚不同所致，结节实际上并没有真正增长。如果依据此变化率确定其为恶性结节，则会造成假阳性。

总之，重建算法及层厚对肺结节容积测量结果有影响，尤其是对直径较小的结节。在未定性肺结节尤其是肺小结节的随访过程中，建议应用相同的重建参数。
